# Physiological Conditions, Bioactive Ingredients, and Drugs Stimulating Non-Shivering Thermogenesis as a Promising Treatment Against Diabesity

**DOI:** 10.3390/ph18091247

**Published:** 2025-08-22

**Authors:** Diego Salagre, Ciskey V. Ayala-Mosqueda, Samira Aouichat, Ahmad Agil

**Affiliations:** 1 Department of Pharmacology, School of Medicine, University of Granada, 18016 Granada, Spain; dsalagre@ugr.es (D.S.); ciskey@correo.ugr.es (C.V.A.-M.); s.aouichat.phd@gmail.com (S.A.); 2 Nutrition, Metabolism, Growth and Development Group, BioHealth Institute Granada (ibs.GRANADA), 18012 Granada, Spain; 3 Neuroscience Institute “Federico Olóriz”, Biomedical Research Center (CIBM), University of Granada, 18016 Granada, Spain; 4 Team of Cellular and Molecular Physiopathology, Faculty of Biological Sciences, University of Sciences and Technology Houari Boumediene, El Alia, Algiers 16111, Algeria

**Keywords:** obesity, diabetes mellitus, type 2, thermogenesis, adipose tissue, brown, muscle, skeletal, food bioactive ingredients, melatonin, cold exposure, exercise, fasting

## Abstract

Obesity (lipotoxicity) results from a chronic imbalance between energy intake and expenditure. It is strongly associated with type 2 diabetes mellitus (T2DM, glucotoxicity) and considered a major risk factor for the development of metabolic complications. Their convergence constitutes “diabesity”, representing a major challenge for public health worldwide. Limited treatment efficacy highlights the need for novel, multi-targeted therapies. Non-shivering thermogenesis (NST), mediated by brown and beige adipose tissue and skeletal muscle, has emerged as a promising therapy due to its capacity to increase energy expenditure and improve metabolic health. Also, skeletal muscle plays a central role in glucose uptake and lipid oxidation, further highlighting its relevance in diabesity. This review explores current and emerging knowledge on physiological stimuli, including cold exposure, physical activity, and fasting, as well as bioactive ingredients and drugs that stimulate NST in thermogenic tissues. Special emphasis is placed on melatonin as a potential regulator of mitochondrial function and energy balance. The literature search was conducted using MEDLINE and Web of Science. Studies were selected based on scientific relevance, novelty, and mechanistic insight; prioritizing human and high-quality rodent research published in peer-reviewed journals. Evidence shows that multiple interventions enhance NST, leading to improved glucose metabolism, reduced fat accumulation, and increased energy expenditure in humans and/or rodents. Melatonin, in particular, shows promise in modulating thermogenesis through organelle-molecular pathways and mitochondrial protective effects. In conclusion, a multi-target approach through the activation of NST by physiological, nutritional, and pharmacological agents offers an effective and safe treatment for diabesity. Further research is needed to confirm these effects in clinical practice and support their use as effective therapeutic strategies.

## 1. Introduction

**Figure 1:**
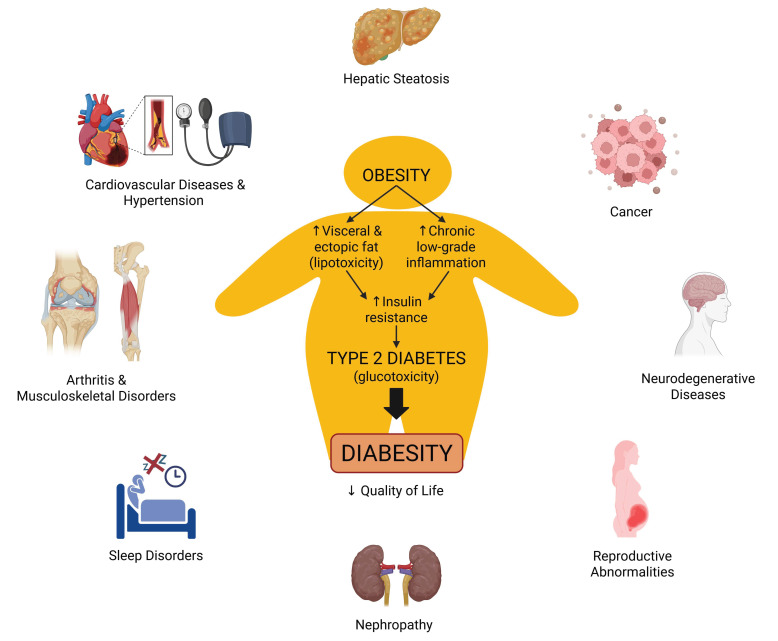
The most common clinical conditions that are associated with obesity and type 2 diabetes (diabesity), and their complex relationship, which directly impact the quality of life of patients.

Modern lifestyle trends in our current society, characterized by abundant calorie intake, lower levels of physical activity, and longer life expectancy, have contributed to a higher prevalence of obesity (mainly associated with visceral fat accumulation), propelling it to an alarming worldwide expansion [1]. According to recent data published by the World Health Organization, the global prevalence of obesity has tripled. In 2022, more than 2.5 billion adults (aged 18 years and older, 43%) were overweight (body mass index (BMI) ≥ 25 kg/m^2^), and 890 million among these (16%) were obese (BMI ≥ 30 kg/m^2^) [2]. Obesity causes numerous physiological dysfunctions that affect every organ and system, producing multiple comorbidities such as type 2 diabetes mellitus (T2DM), also called diabesity (obesity and its associated T2DM). Diabesity increases the risk of cardiovascular diseases, hepatic steatosis, nephropathy, arthritis, musculoskeletal disorders, neurodegenerative diseases, certain types of cancers, and reproductive abnormalities (Figure 1). Furthermore, obese people have a lower quality of life, indicating a psychosocial impact as well. The etiology of obesity is multifactorial, involving a complex interaction among genetics, physiology, behavior, and the environment. Environmental factors lead to the prevalence of poor eating habits, such as having facilities where one can obtain unhealthy food in close proximity with very reduced prices, and the sedentary lifestyle that prevails in a great part of the population [3]. In line with the previously mentioned facts, developing efficient treatment approaches to address obesity is crucial. However, current interventions based on lifestyle changes and increased physical activity often fail to produce consistent results, with success achieved in only a small proportion of individuals [3,4]. Hence, effective and safe therapeutic strategies are urgently required to curb the rising prevalence of obesity. Targeting energy expenditure through thermogenesis activation is considered a new, promising approach against diabesity and its related metabolic disorders [4].

Thermogenesis is a physiological mechanism where energy is dissipated as heat for whole-body temperature maintenance in mammals [5]. It is divided into shivering thermogenesis, induced by repetitive muscle contraction, and non-shivering thermogenesis (NST) in skeletal muscle and thermogenic adipose tissues [4]. Brown adipose tissue (BAT) and beige adipose tissue (bAT) are rich in multilocular lipid droplets and abundant mitochondria expressing uncoupling protein 1 (UCP1) with great thermogenic function, unlike white adipose tissue (WAT), which is characterized by lipid storage for times of energy need [6]. Both BAT and bAT have been given greater importance as thermogenic organs, despite representing only 0.2% of body weight. However, thermogenic activity in skeletal muscle has been emerging as an attractive strategy, given that this organ accounts for one-third of body weight and has a greater contribution to basal metabolic rate [4]. Indeed, skeletal muscle is the organ that contributes the most to increasing energy expenditure by NST through the uncoupling of the sarcoendoplasmic reticulum calcium ATPase (SERCA) pump induced by sarcolipin (SLN) binding [4,5].

Several reviews have previously addressed thermogenesis as a therapeutic strategy against obesity and related metabolic disorders. For example, Cheng et al. [6] focused primarily on the role of brown and beige adipose tissue, while Li et al. [4] explored NST specifically in skeletal muscle, but without integrating both tissues’ thermogenesis. Betz and Enerbäck [5] emphasized physiological thermogenic stimuli such as cold and the thermogenic molecular mechanisms in adipose and muscle tissue, offering neither a multi-target therapeutic perspective nor a translational point of view. Although these studies have advanced our understanding, none has provided a comprehensive and complete analysis encompassing physiological stimuli (cold exposure, exercise, and fasting), bioactive compounds (many of which come from foods and spices that can be integrated into the diet), and pharmacological agents—including the emerging role of melatonin—while taking into account the thermogenic molecular pathways involved in both adipose and muscle tissues in the context of diabesity. This review addresses this gap by combining physiological, nutritional, and pharmacological approaches from a clinical perspective, proposing their combined use, and encouraging further research into their possible synergistic effects in improving glucose and lipid metabolism. By unifying these perspectives, the current review aims to summarize both established and emerging findings regarding the physiological conditions, bioactive agents, and drugs that stimulate NST with potential implications for the control of obesity and diabetes management. Particular emphasis is placed on melatonin as a novel and promising thermogenic agent to combat diabesity.

## 2. Methods

To provide a comprehensive overview of current and emerging strategies that stimulate NST with potential anti-diabesity effects, this review focused on physiological conditions, bioactive compounds, and pharmacological agents that promote thermogenic activation in brown/beige adipose tissue and skeletal muscle. The literature search was conducted in May 2025 using MEDLINE via PubMed and Web of Science databases. The following keywords and MeSH terms were used in various combinations: Obesity; Diabetes Mellitus, Type 2; Thermogenesis; Adipose Tissue, Brown; Muscle, Skeletal; Food Bioactive Ingredients; Melatonin; Cold Exposure; Exercise; Fasting; Capsaicin; Paradol; Gingerol; Zingerone; Cinnamaldehyde; Cinnamic acid; Curcumin; Allicin; Quercetin; Caffeine; Catechin; Theaflavin; Flavanol; Berberine; Docosahexaenoic acid; Omega-3 polyunsaturated fatty acid; Eicosapentaenoic acid; Eriocitrin; Menthol; Thymol; Resveratrol; Proanthocyanidin; Anthocyanin; Mirabegron; Levothyroxine; Liothyronine; Triiodothyronine; Resmetirom; Fexaramine; Farnesol; Chenodeoxycholic acid; Growth Hormone; Somatotropin; Tesamorelin; Liraglutide; Semaglutide; Exedin-4; Tirzepatide; Oxyntomodulin; Mazdutide; Retatrutide; Dapagliflozin; Empagliflozin; and Canagliflozin.

Additional relevant articles were identified by manually screening the reference lists of key papers and recent reviews. No date restrictions were applied to allow inclusion of pioneering studies and foundational discoveries, regardless of publication year. Articles were selected based on their scientific relevance, novelty, methodological robustness, and depth of mechanistic insight, with priority given to studies in humans and, when not available, to well-designed rodent studies. Only peer-reviewed original articles, systematic reviews, and narrative reviews written in English were considered. Studies in both in vivo and in vitro settings were included if they provided significant insight into NST activation. Exclusion criteria included non-peer-reviewed materials, case reports, conference abstracts, and older articles that lack mechanical relevance to the scope of the review or contain data corroborated in more recent studies. All selected papers were independently screened and discussed by the authors to ensure inclusion of the most informative and impactful literature. No formal quality assessment tool was applied, as this was a narrative review aiming to synthesize high-quality, conceptually relevant evidence across a broad timespan.

All figures and illustrations were created by the authors using the Biorender graphic tool (https://www.biorender.com/, accessed on 15 June 2025), and the molecular structures of the compounds were obtained using the PubChem tool (https://pubchem.ncbi.nlm.nih.gov/, accessed on 12 August 2025).

## 3. White and Thermogenic Adipose Tissues

**Figure 2:**
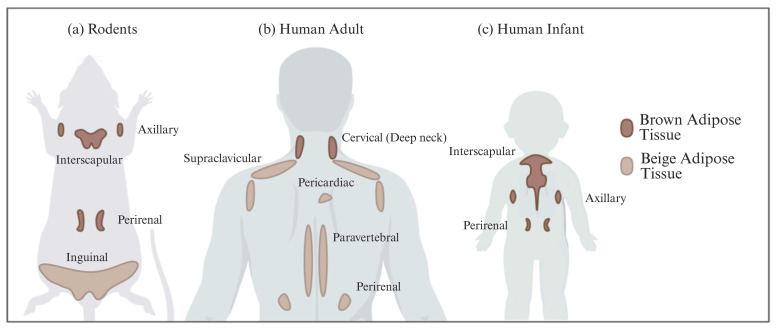
Topographic anatomical locations of thermogenic adipose depots in (**a**) rodents, (**b**) human adults, and (**c**) human infants. Human infants classically have mainly brown adipose tissue (BAT), located in similar locations as rodents, such as axillary, perirenal, and most importantly, interscapular. Rodents additionally contain a large white adipose tissue (WAT) depot with recruitable beige fat in the inguinal region. Adult humans have few classical BAT depots, such as cervical (deep neck), but a larger proportion of beige adipose tissue (bAT) has been found in the supraclavicular, pericardiac, paravertebral, and perirenal regions.

In all mammals, including humans, three different types of adipose depots can be found, known as white, brown, and beige adipose tissues. They have unique characteristics that differentiate them from each other in terms of function, morphology, structure, protein expression profile, and developmental origin. WAT can also be differentiated into visceral WAT (vWAT), or abdominal fat, which is distributed around internal organs and is located inside the peritoneum; and subcutaneous WAT (sWAT), which is located underneath the skin. vWAT tends to cause low-grade inflammation that promotes insulin resistance and is responsible for the morbidity associated with obesity, whereas sWAT is metabolically active with less inflammatory activity, and could favor metabolic insulin action [7]. The function of WAT is to store excess energy in the form of triglycerides, which can be released to fuel other tissues during fasting periods, whereas BAT plays a crucial role in whole-body energy homeostasis through NST. White adipocytes are unilocular cells with a large lipid droplet and a few mitochondria that are devoid of UCP1 (Table 1). bAT, also known as “inducible” or “recruitable” beige fat, is defined as a cluster of UCP1-expressing and mitochondria-rich adipocytes that, in response to various external stimuli, are developed within the WAT depots. Beige/brite adipocytes, similarly to BAT adipocytes, are recognized for their characteristic lipid morphology consisting of small and multilocular lipid droplets, their high mitochondrial content, and their positive expression of UCP1 (Table 1) [8]. The switch from white adipocyte phenotype to brown-like phenotype and thus bAT recruitment in response to external stimuli is called browning, britening, and/or beiging. Two theories have been proposed by the scientific community regarding the origin of beige adipocytes: (i) de novo differentiation from progenitor cells that reside in WAT, and (ii) reversible transdifferentiation, a bidirectional mechanism that involves interconversion of mature white adipocytes into mature beige/brite adipocytes and vice versa [9]. The most common anatomical location of BAT and bAT in rodents and humans is shown in Figure 2.

**Figure 3:**
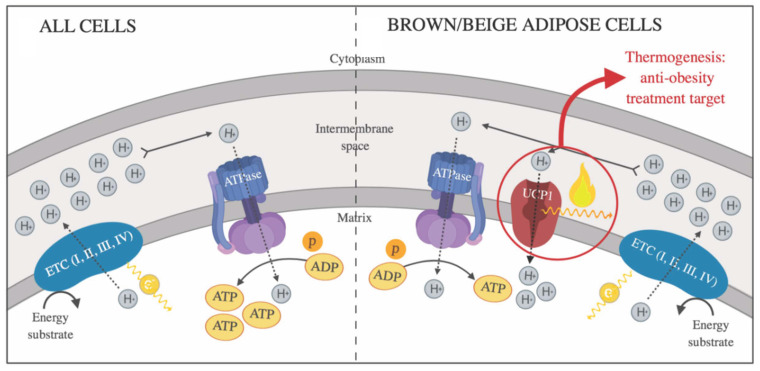
Uncoupling OXPHOS mechanism by UCP1 in thermogenic adipose cells in the mitochondrial intermembrane space. On the left, ATPase pumps protons down their gradient to catalyze the synthesis of ATP in all cells, including WAT cells. On the right, UCP1 in BAT and bAT facilitates a proton leak from the intermembrane space back into the matrix, from which the energy produced is released as heat (thermogenesis). I, Complex I NADH dehydrogenase; II, Complex II succinate dehydrogenase; III, Complex III cytochrome bc1 complex; IV, cytochrome c oxidase; ADP, Adenosine diphosphate; ATP, Adenosine triphosphate; ATPase, Complex V Adenosine triphosphate synthase; ETC, Electron transport gradient; H^+^, Hydrogen ion/proton; P, phosphate; UCP-1, Uncoupling protein-1/Thermogenin.

In mitochondria from BAT and bAT, the transmembrane protein UCP1 is responsible for thermogenic adipose tissue’s contribution to NST. Under certain stimuli, UCP1 is activated and generates heat production by allowing protons to re-enter the mitochondrial matrix, uncoupling substrate oxidation and oxidative phosphorylation (OXPHOS) from the ATP synthesis [10], from which the energy produced is released as heat (Figure 3).

## 4. Skeletal Muscle and NST

**Figure 4:**
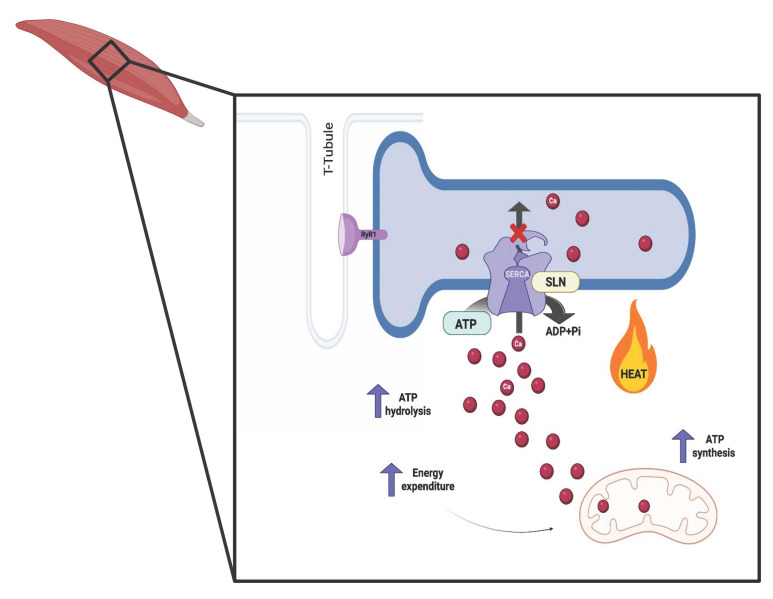
Uncoupling of sarcoendoplasmic reticulum calcium ATPase (SERCA) by sarcolipin (SLN) and crosstalk between sarcoplasmic reticulum and mitochondria in skeletal muscle. Ca, Calcium; Pi, inorganic Phosphate; RyR1, Ryanodine Receptor 1; ADP, Adenosine diphosphate; ATP, Adenosine triphosphate.

Skeletal muscle plays a key role in thermoregulation through the production of heat by non-shivering mechanisms. This organ represents around 50% of total body weight and is responsible for 70–80% insulin-stimulated glucose uptake, fatty acids metabolism, and whole-body energy expenditure through futile cycles [5,11]. In skeletal muscle, NST is activated by the uncoupling of SERCA. The SERCA pump is a transmembrane protein belonging to the family of P-type ATPases, whose main function is the transport of cytosolic calcium back to the sarcoplasmic reticulum using the energy generated from ATP hydrolysis (Figure 4), thus regulating calcium concentrations in both spaces following muscle contraction [12]. SERCA function is regulated by endogenous molecules such as SLN [11]. SLN is a single-spanning membrane protein, the expression of which predominates in skeletal muscle, and its expression increases with overloading and exercise performance in mice [13]. By dissociating calcium (Ca^2+^) ions from SERCA activity, it regulates muscle NST, allowing SERCA to repeatedly run a futile cycle of ATP hydrolysis into ADP without efficiently pumping Ca^2+^ ions into the endoplasmic reticulum. This results in the dissipation of the excess of energy not used for Ca^2+^ transport in the form of heat (Figure 4), playing an essential role in maintaining body temperature and representing another critical function of muscle beyond contraction [12]. This thermogenic mechanism used by skeletal muscle is indirectly associated with mitochondrial metabolism (Figure 4), unlike BAT, where thermogenic activity directly depends on mitochondrial respiration and metabolism [14].

## 5. Physiological Conditions Stimulating NST

### 5.1. Cold Exposure

Cold is a powerful natural stimulus for the activation of adaptive thermogenesis, thus generating heat production in BAT and skeletal muscle. It is well known that cold exposure increases energy expenditure and reduces insulin resistance, making it an interesting strategy during diabesity treatment [15]. Several studies in rodents have demonstrated that both acute and chronic cold activate thermogenic activity in BAT and lead to the emergence of UCP1-positive cells, as well as morphological changes in rodents’ white fat pads [16]. For example, Lim et al. [17] demonstrated an increase in UCP1-positive mitochondria in mice’s sWAT in response to prolonged cold exposure under 4 °C. In addition to molecular changes, the acquisition of brown-like adipose phenotype in WAT, simultaneously with increased vascular density, owing to activation of angiogenesis, was markedly observed. Activation of BAT thermogenesis was also noted in this study. In humans, BAT was shown to be effectively recruited and activated upon cold acclimation. For instance, one study showed that mild cold acclimation for 10 days in healthy subjects performing sedentary activities was enough to recruit BAT and increase NST but failed to promote browning of abdominal WAT [18]. Even in an obese man with low BAT activity, short-term cold acclimation increased cold-induced glucose uptake in BAT [18]. A study conducted in humans has shown that BAT can be successfully activated and recruited through cold acclimation; however, only one of the human studies with 4 °C cold chronically found WAT browning [19], suggesting that moderate acute cold exposure may not be sufficient to promote WAT browning in humans. In muscle tissue, studies in mice have shown that cold upregulates SLN expression and decreases SERCA pump activity, thus producing heat [20]. Hanssen et al. [21] showed in a ten-day study that acute cold exposure increased insulin sensitivity in the skeletal muscle of patients with type 2 diabetes through GLUT4 upregulation in the sarcolemma responding to cold acclimation. Changes in the mitochondrial architecture of muscle from mice exposed to mild cold (16 °C) and severe cold (4 °C) were also observed with ultrastructural remodeling of mitochondria cristae, especially in severe cold [14]. In addition, skeletal muscle and BAT thermogenesis have been proposed to be regulated by circulating myokines, as a close crosstalk between both processes was identified. Rowland et al. used SLN-Knockout (KO), UCP1-KO, and double-knockout (DKO) mice and exposed them gradually to cold. The lack of SLN or UCP1 was compensated for with an increase in the expression of the other protein after cold adaptation. DKO mice were affected by acute cold, developing hypothermia, consequently increasing food intake, and mobilizing almost all of their white fat. Therefore, cold exposure without SLN and UCP1 represented a high energy cost and the use of unsustainable thermogenic mechanisms [22].

The mechanism of cold-induced BAT and skeletal muscle non-shivering thermogenesis is mostly elucidated in small rodents. Briefly, cold is sensed by thermal transient receptor potential channels (TRPs), which are proteins that detect environmental changes, such as temperature, pain, touch, osmolarity, and certain natural compounds. When cold-activated TRPs in skin sensory neurons send signals to the brain, sympathetic nerve activity increases. Noradrenaline released from sympathetic terminals then triggers NST activation in BAT and skeletal muscle, as well as brown-like phenotype in WAT or browning through β3-adrenergic receptor (β3-ARs) activation and peroxisome proliferator-activated receptor gamma (PPARγ) coactivator 1-alpha (PGC1α) expression (the key regulator of thermogenesis) [12,16].

### 5.2. Physical Exercise

Physical exercise itself generates heat through fiber contraction, like cold-induced shivering thermogenesis. However, there is also a close relationship between exercise and non-shivering thermogenesis due to circulating myokines released after exercise. Irisin is the most important exercise-induced adipomyokine, which is involved in the activation of NST by inducing BAT activation and browning of white fat cells in both rodents and humans [23]. When performing physical exercise, PGC1α is activated in skeletal muscle, enhancing mitochondrial biogenesis and inducing the synthesis of a membrane protein named fibronectin-type III domain-containing 5 (FNDC5) expressed in skeletal muscle as well. The FNDC5 N-terminal end is released into the blood, and this product is known as irisin. Thus, sedentary people have lower levels of irisin in their blood compared to athletes or individuals engaged in exercise [23,24]. Nonetheless, Stengel et al. reported that in obese patients, irisin levels are altered as a possible physiological response to improve insulin resistance in these patients [25]. Transgenic mice overexpressing PGC1α in skeletal muscle were studied, and the results showed not only an enhanced and preserved skeletal muscle mitochondrial function but also an improvement in insulin sensitivity and glucose homeostasis [26].

In humans, combined training increases mitochondrial enzyme activity and activates BAT in obese and diabetic patients [27], and Boström’s group showed that irisin, the exercise-induced muscle-derived protein, plays a critical role in browning of WAT [24]. In their study, they found that irisin acts to stimulate UCP1 expression and a wide program of brown fat-like development. Similar effects were found in rodents subjected to an exercise program, after which elevated irisin levels in blood and adipose tissue, and subsequently increased expression of WAT browning markers, were observed [28]. Moreover, a recent study in a rat model of postmenopausal obesity showed that irisin supplementation not only improved the adiposity of vWAT and increased BAT and bAT activation and recruitment by browning but also increased SERCA1 and SLN expression, showing that irisin also activates skeletal muscle NST [29]. In addition, irisin was shown to play an important role in glucose sensitivity and lipid metabolism in skeletal muscle [23]. Also, irisin release after fiber contraction increased the expression of UCP3 mRNA in skeletal muscle [30] and was proposed to be essential for adaptive thermogenesis to extreme cold by promoting skeletal muscle mitochondrial biogenesis through UCP3 regulation and BAT activation.

### 5.3. Fasting

Fasting is an increasingly popular dietary approach in which the consumption of energy-providing foods and beverages is restricted for a certain number of hours. A wealth of studies highlight the efficacy of fasting in the prevention of obesity, which goes beyond thermogenesis activation and weight loss [31], as fasting also provides numerous health benefits by improving insulin sensitivity and metabolic alterations [32]. Although the results are promising for the use of fasting to reduce body weight and improve metabolic health, studies aimed at understanding the mechanism underlying these benefits in humans are scarce, with the gut microbiome being proposed as the main axis orchestrating the whole process [33]. Putri et al. also proposed CD36, a protein that participates in the transport of fatty acids in skeletal muscle and adipose tissue, as the key protein involved in thermogenesis and fasting regulation. CD36-KO mice subjected to fasting and cold exposure showed marked hypothermia due to energy depletion in BAT and skeletal muscle, and limited nutrient supply to these tissues [34].

Li et al. [33] demonstrated for the first time that intermittent fasting (IF) increases the total energy expenditure and selectively induces WAT browning by re-shaping gut microbiota, which led to the elevation of browning stimuli via increased short-chain fatty acid (SCFA) release, such as acetate and lactate. Similar results were obtained in rodents in which fasting was shown to activate PGC1α and UCP1 expression in WAT, reducing the adipocyte size and switching its morphology and function (browning), and in BAT [35,36]. In addition, IF increases energy expenditure and promotes BAT activation and sWAT browning in rodents under high-fat diet (HFD)-induced obesity [37]. Beyond the direct effects of fasting on thermogenesis, IF has been shown to induce changes in the gut microbiota, restoring it in obese patients, who had clear dysbiosis compared to healthy subjects [38]. The gut microbiota plays a crucial role in metabolic homeostasis by influencing nutrient sensing and absorption, hormonal regulation, and redox balance, thereby modulating key metabolic signaling pathways and overall energy balance [39]. Environmental factors such as ambient temperature have been associated with modifications in the microbiota of rodents, highlighting the close relationship between adaptive thermogenesis and the gut microbiota [40]. Depletion of the gut microbiota via antibiotics and germ-free mice was found to present reduced thermogenic capacity and UCP1 expression [41]. Butyrate supplementation, another SCFA produced by the gut microbiota, reversed these effects, increasing the thermogenic capacity and heat production and restoring the UCP1 expression in WAT and BAT in mice [41], underscoring the role of microbial metabolites in adaptive thermogenesis. IF also improved insulin resistance by modulating the gut microbiota and bile acid metabolism in diet-induced obese mice [42]. Furthermore, it has been proposed that the gut microbiota itself acts as a “thermogenic organ”, contributing up to 8% of resting energy expenditure through anaerobic fermentation processes [43]. These findings highlight thermogenic microbiota as an emerging and underexplored pathway in energy balance regulation, potentially acting as an indirect modulator of fasting-induced thermogenesis. Further studies will help to determine its translational relevance and potential use in clinical practice in the fight against diabesity.

The molecular mechanisms underlying the effect of IF on BAT thermogenesis and WAT browning are still unclear, but activation of β3-ARs mediated by Sirtuin 6 (SIRT6) may be essential in the regulation of these processes [44]. In humans, IF failed to alter UCP1 mRNA levels in sWAT from obese and overweight women [37]. However, an increase in skeletal muscle *UCP3* gene expression was observed after 40 h of fasting [45]. Data from rodents propose IF as a novel strategy for BAT activation, beige fat development, and treatment of metabolic diseases, although further clinical investigations with well-designed protocols are needed to clarify its effectiveness in humans.

**Figure 5:**
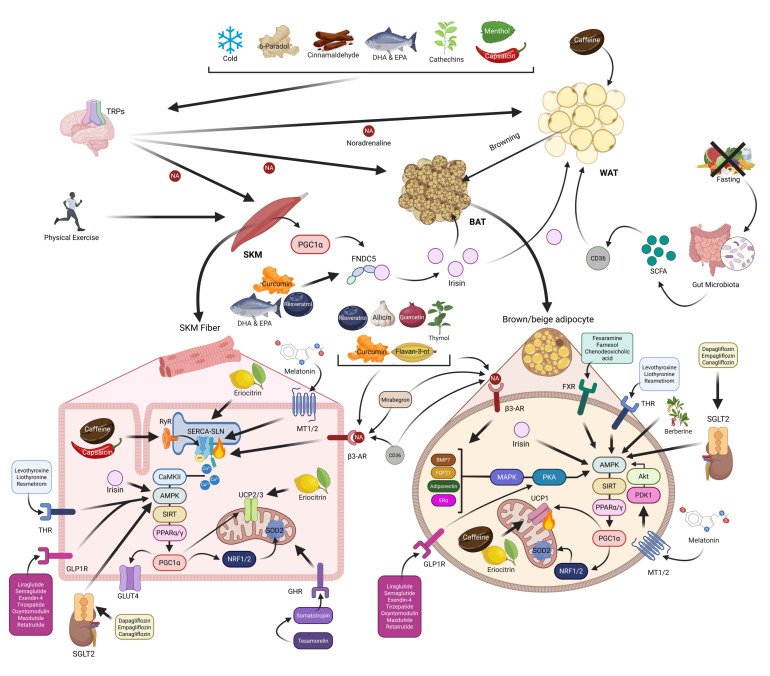
Physiological conditions, bioactive agents, and drugs stimulating non-shivering thermogenesis (NST). Akt, protein kinase B; AMPK, 5′ adenosine monophosphate-activated protein kinase; ATP, adenosine triphosphate; β3-AR, β3 adrenergic receptor; BAT, brown adipose tissue; BMP7, bone morphogenetic protein 7; Ca^2+^, calcium; CaMKII, Ca^2+^/calmodulin-dependent protein kinase II; CD36, cluster of differentiation 36; DHA, docosahexaenoic acid; EPA, eicosapentaenoic acid; ERα, estrogen receptor α; FGF21, fibroblast growth factor 21; FNDC5, fibronectin type III domain-containing protein 5; FXR, farnesoid X receptor; GHR, growth hormone receptor; GLP1R, glucagon-like peptide-1 receptor; GLUT4, glucose transporter type 4; MAPK, mitogen-activated protein kinases; MT1/2, melatonin receptor 1/2; N, nitrogen; NA, noradrenaline; NRF1/2, nuclear respiratory factor 1/2; O, oxygen; PDK1, pyruvate dehydrogenase kinase 1; PGC1α, peroxisome proliferator-activated receptor gamma co-activator 1α; PKA, protein kinase A; PPARα/γ, peroxisome proliferator-activated receptor α/γ; RyR, ryanodine receptor; SCFA, short-chain fatty acids; SERCA, sarcoplasmic/endoplasmic reticulum Ca^2+^-ATPase; SGLT2, sodium-glucose linked transporter 2; SIRT, sirtuin; SKM, skeletal muscle; SLN, sarcolipin; SOD2, superoxide dismutase 2; T3, triiodothyronine; THR, thyroid hormone receptor; TRP, transient receptor potential; UCP1, uncoupling protein 1; UCP2/3, uncoupling protein 2/3; WAT, white adipose tissue.

This section highlights the significant influence of physiological conditions such as cold exposure, physical exercise, and fasting on the activation of NST through mechanisms involving BAT, skeletal muscle, and WAT browning (Table 2 and Table 3 and Figure 5). While promising results have been observed, particularly in rodent models, the translation to human physiology remains inconsistent and requires further investigation.

## 6. Bioactive Ingredients and Their Role in Promoting NST

Several bioactive compounds have been shown to promote NST through BAT activation/WAT browning and/or skeletal muscle SERCA–SLN uncoupling. Food-derived bioactive agents have been extensively studied for their potential in treating diabesity due to their positive health effects.

**Capsaicin** (C_18_H_27_NO_3_), the primary capsaicinoid from chili peppers and their major pungent compound, is widely known for its thermogenic and anti-obesity effects. In skeletal muscle, capsaicin has been proposed to be a SERCA activity regulator and, thus, an activator of skeletal muscle NST, as it increases SERCA, the ryanodine receptor (RyR), and UCP2/3 expression in C2C12 myotubes [46]. Regarding thermogenic adipose tissue activation, oral ingestion of capsinoids has been shown to increase BAT activity, abdominal fat loss, and fat oxidation in humans [47]. In rodents, capsinoids also stimulate WAT browning through TRP Vanilloid 1 (TRPV1) activation in the gastrointestinal tract [48], suggesting an activation of the vagal afferent nerves that project into the ventromedial hypothalamus, stimulating the sympathetic nervous system and catecholamine secretion via β3-ARs. This enhances whole-body energy expenditure and reduces fat content via calcium/calmodulin-activated protein kinase II (CaMKII)/AMP-activated kinase (AMPK)/SIRT1/PGC1α signaling pathway activation, leading to the expression of thermogenic markers like UCP1 and PRDM16 [48].

**6-Paradol** (C_17_H_26_O_3_), found in ginger and the Grain of Paradise (GP), is another TRPV1 agonist with thermogenic properties. In humans, the GP extract increased whole-body energy expenditure and BAT function, thereby reducing visceral fat [49,50]; however, ginger’s thermogenic effects remain limited, with no evidence regarding energy expenditure. In obese mice, diets enriched in different ginger extracts promoted energy expenditure and lipolysis, suppressed adipogenesis in WAT, and stimulated BAT activity, increasing UCP1 expression and oxygen consumption [51]. In mice, ginger and its major components, **6-Gingerol** (C_17_H_26_O_4_) and **Zingerone** (C_11_H_14_O_3_), were also found to increase heat production by WAT browning [52,53] and muscle mitochondrial biogenesis [54]. The mechanisms underlying the thermogenic effects of ginger may be partly regulated by the SIRT1/AMPK/PPARα/PGC1α pathway [52,54], possibly via β3-ARs activation.

**Cinnamaldehyde** (C_9_H_8_O), the pungent compound in cinnamon, has been shown to protect rodents against HFD-induced obesity, enhancing body temperature, BAT function, and WAT browning through increased mitochondrial ATP production and UCP1, PGC1α, PPARγ, PRDM16, and fibroblast growth factor 21 (FGF21) expression [55,56]. The effects were attributed to cinnamaldehyde-induced adrenaline secretion via TRPA1 and thus β3-ARs activation [57]. In adipocytes derived from human subcutaneous fat [57] and in healthy subjects, cinnamaldehyde increased energy expenditure and postprandial fat oxidation [58]; however, more clinical trials are needed to further elucidate the thermogenic effects. Other cinnamon-derived compounds, like **Cinnamic Acid** (C_9_H_8_O_2_), also promote fat browning and thermogenic activity by activating β3-ARs and AMPK signaling in mice [59].

**Curcumin** (C_21_H_20_O_6_), a polyphenol from turmeric, has been linked to anti-obesity effects, increasing energy expenditure and reducing fat mass and waist/hip circumference in overweight individuals [60]. It modulates SERCA activity in dynamic muscle cells from rodents [61], regulating muscle function and NST. In addition, in mice, curcumin improves plasma myokine levels of FNDC5 and irisin, insulin sensitivity, energy expenditure, oxygen consumption, and heat production, accompanied by white adipocyte and WAT browning, BAT and sWAT metabolic activity enhancement, and increased UCP1 expression [62]. These thermogenic effects are regulated via AMPK upregulation and noradrenaline secretion, suggesting the activation of a β3-ARs-dependent pathway [63].

**Allicin** (C_6_H_10_OS_2_), an organosulfur compound found in garlic, has antioxidant and immunomodulatory properties with strong potential in the treatment of several diseases, such as diabetes mellitus, cardiovascular diseases, and cancer. In obese mice, allicin administration enhances weight loss and thermogenesis by promoting lipolysis and increasing UCP1, PGC1α, and PRDM16 expression in both BAT and WAT, possibly through Sirtuins-mediated PPARα cascade activation [64]. Garlic administration to obese rodents also showed enhanced UCP expression in BAT, WAT, and skeletal muscle via β3-AR and AMPK-dependent pathway activation [65]. In addition, a fermented garlic extract was also found to increase UCP1 expression in human adipose-derived stem cells, increasing cell metabolism, fat oxidation, and mitochondrial oxygen consumption [66].

**Quercetin** (C_15_H_10_O_7_) is a polyphenol flavonoid found mainly in onions. In rodents, quercetin was also found to increase weight loss, energy expenditure, and heat production, not only by enhancing BAT mass and function but also by increasing WAT browning and modulating UCP1, PGC1α, and PPARγ expression [67]. In vitro, quercetin-rich extracts enhanced brown and beige-specific gene expression and increased lipolysis, fat β-oxidation, and glucose uptake through PPARα, FGF21, AMPK [68], and β3-ARs stimulation [67]. Moreover, quercetin and its derivatives were also known to modulate SERCA activity and function in muscle from rodents, showing its potential role in skeletal muscle NST directly binding to the ATPase domain [69], which may modulate mitochondriogenesis, energy expenditure, fat oxidation, mitochondrial function, adiposity, and insulin sensitivity.

**Caffeine** (C_8_H_10_N_4_O_2_), a psychostimulant consumed mainly in coffee, tea, and cocoa beans, has thermogenic effects on skeletal muscle, increasing energy expenditure and muscle heat production. In addition, a study suggested that 60 mg/kg of caffeine upregulates the expression of UCP1 in BAT and UCP3 in skeletal muscle of obese mice [70]. In humans, supraclavicular BAT was activated after acute caffeine administration, enhancing heat production and energy expenditure [71]. Also, in mice, caffeine promotes browning of white adipocytes to beige-like adipocytes and de novo differentiation of brown adipocytes [71], increasing UCP1, PPARγ, PGC1α, and PRDM16 expression and mitochondrial biogenesis [72]. The mechanisms underlying caffeine-induced skeletal muscle NST are still unclear, whereas the observed thermogenic actions are suggested to be mediated by calcium transport uncoupling, as caffeine is a RyR agonist.

Similar to caffeine, **Catechins** from tea, such as epigallocatechin-3-gallate (C_22_H_18_O_11_), are associated with weight loss and increased energy expenditure. In white adipocytes from mice, catechins increased lipid metabolism and heat production through UCP1 upregulation, promoting direct white-to-brown adipocyte transformation [73]. In rodents, green tea extracts and catechins promote body weight reduction and enhanced energy expenditure by BAT activation and/or WAT browning, increasing UCP1 expression through adiponectin-dependent PPARγ, PGC1α, PRDM16, bone morphogenetic protein 7 (BMP7), and FGF21 activation [74]. In humans, catechin beverages increased energy expenditure through direct activation of TRP Anquirina 1 (TRPA1) and TRPV1 in the sensory neurons of the gastrointestinal tract, and consequently activation and/or recruitment of BAT [75]. In addition, black tea **Theaflavins** (C_29_H_24_O_12_) in mice also increased muscle UCP3 through the AMPK/PGC1α activation pathway [76].

**Flavan-3-Ols** (C_15_H_14_O_2_), flavonoids found in cocoa beans, nuts, berries, stone fruits, apples, pears, and also green tea, increase UCP3 and PGC1α expression in the skeletal muscle from mice, enhancing total energy expenditure [77,78]. In rodents, cocoa flavonols increased BAT activation through AMPK/β3-ARs activation, increasing UCP1 and PGC1α expression [78].

**Berberine** (C_20_H_18_NO_4_^+^), a polyphenolic compound from herbs like *Coptis chinensis* (Chinese goldthread) and *Hydrastis canadensis* (goldenseal), has been reported to exert anti-diabetic and anti-hyperlipidemia effects in humans. In obese and diabetic mice, berberine increased BAT activity, WAT browning, and mitochondrial content through AMPK/SIRT1/PPARγ pathway activation and thermogenic marker upregulation (UCP1 and PGC1α), as recently reviewed in [79]. Berberine also promoted brown adipocyte differentiation in both mice and human primary preadipocytes [80]. In humans, chronic berberine treatment promoted weight loss and increased BAT mass and activity in mildly overweight subjects [80].

**Docosahexaenoic acid** (DHA, C_22_H_32_O_2_), an Omega-3 polyunsaturated fatty acid (PUFA) primarily found in fatty fish and marine algae, has been shown to reduce the efficiency of SERCA activity in rats, suggesting an uncoupling effect similar to SLN [81]. Moreover, DHA increased UCP1 expression in BAT and sWAT, enhancing mitochondrial functionality and energy expenditure in rodents [82]. Similar to DHA, **Eicosapentaenoic acid** (EPA, C_20_H_30_O_2_) in mice also enhances BAT thermogenic properties, increasing PRDM16, PGC1α, and UCP1 expression, and also promotes sWAT browning [82] and muscle NST through SERCA2b and FGF21 upregulation [83], supporting the idea that DHA and EPA could be attractive food-derived bioactive agents to curb obesity. In cultured subcutaneous adipocytes from overweight subjects, EPA induced beige-like adipocytes through AMPK/SIRT1/PGC1α thermogenic pathway activation [84]. The thermogenic effects of PUFAs are proposed to be mediated through the activation of TRPV1 and β3-ARs, as well as browning-related myokines (irisin and FGF21), via muscle calcium signaling [85,86]. All together, these findings suggest a novel and promising role of fish oils (DHA and EPA) in preventing obesity via BAT activation, browning, and skeletal muscle calcium SERCA uncoupling, although their effects on humans are still inconclusive.

**Eriocitrin** (C_27_H_32_O_15_), a flavonoid found in the skin of limes and lemons, has been linked to health benefits. Eriocitrin boosted several thermogenic genes’ expression in mice, such as *UCP1* in BAT and *UCP3*, *SLN*, *SERCA1*, and *SERCA2* in skeletal muscle, enhancing insulin sensitivity, fatty acid oxidation, and energy expenditure and reducing hepatic gluconeogenesis and steatosis [87].

**Menthol** (C_10_H_20_O), a cooling and flavoring alcohol found in mint oils, is a TRPM8 receptor agonist (β3-ARs activation) that has been shown to promote UCP1-dependent BAT thermogenesis, sWAT browning, glucose metabolism, and muscle energy expenditure in mice after chronic supplementation [88,89]. In rodents, menthol administration enhanced hyperthermia through increased UCP1, PGC1α, PRDM16, FGF21, AMPK, and Protein kinase A (PKA) expression [88]. In humans, menthol increases hyperthermia and energy expenditure, showing its potential to promote thermogenesis [90].

**Thymol** (C_10_H_14_O), a monoterpene polyphenolic compound found in thyme, enhances mitochondrial biogenesis and UCP1 expression through β3-ARs and PKA/AMPK signaling pathway activation in mice-derived white adipocytes [91]. These results suggest that thymol may act as a selective β3-sympathomimetic; however, in vivo studies are lacking.

**Resveratrol** (C_14_H_12_O_3_), a polyphenol primarily found in the skin of grapes and other berries, has anti-obesity effects, enhancing thermogenic tissue function. In vitro studies showed that resveratrol upregulates FNDC5/irisin expression in mice [92]. In rodents, resveratrol increased UCP3 expression in skeletal muscle, UCP1 expression in BAT and sWAT, and promoted mitochondrial dynamics, insulin sensitivity, glucose uptake, weight loss, and visceral fat mass loss [93,94]. In rodents, resveratrol decreased adipogenesis and enhanced angiogenesis, mitochondrial activity, and brown-like adipocyte marker expression via BMP7/Estrogen Receptor α (ERα)/AMPK/PPARα/γ/PGC1α activation [93,95]. In humans, resveratrol-enriched diets increased UCP1, PRDM16, PGC1α, and SIRT1 expression in sWAT biopsies, improving the glycemic and lipid profiles of individuals through FNDC5 activation [93]. Additionally, other dietary sources of resveratrol, like berry extracts [96,97], also showed similar thermogenic effects on BAT activity and/or WAT browning in rodents. Similarly, **Proanthocyanidin** (C_31_H_28_O_12_) and **Anthocyanin** (C_15_H_11_O^+^), other grape-derived bioactive compounds, also were shown to promote energy expenditure through increased skeletal muscle and BAT mitochondrial function [98], BAT activity, and sWAT browning in rodents [99]. All these compounds activate the β3-ARs pathway and increase the expression of thermogenic markers such as UCP1, PRDM16, and PGC1α, thereby limiting obesity [100].

Despite compelling evidence supporting the thermogenic effects of these food-derived bioactive compounds (Table 2 and Table 3 and Figure 5), human studies remain limited or very scarce, and further research is needed to elucidate their potential in anti-diabesity therapies.

## 7. Drugs Stimulating NST

### 7.1. FDA-Approved Drugs

Although numerous drugs exist for obesity-related conditions such as diabetes and cardiovascular diseases, pharmacological interventions specifically targeting obesity remain limited. Currently, only a few FDA-approved drugs are available for long-term obesity management, primarily acting by suppressing appetite or inhibiting lipid absorption. In contrast, therapies that enhance adaptive thermogenesis—particularly via BAT activation or WAT browning—are still underexplored [101].

**β3-ARs agonists** have emerged as good pharmacological tools to fight obesity and its complications due to their thermogenic effects. β3-ARs are abundant in thermogenic tissues such as BAT, regulating NST through sympathetic nerve stimulation and enhancing lipolysis, energy expenditure, and weight loss via AMPK/SIRT1/PGC1α pathway activation and UCP1 upregulation [101]. The most studied β3-ARs agonist is Mirabegron (C_21_H_24_N_4_O_2_S), an FDA-approved drug for overactive bladder syndrome. In rodents, mirabegron was shown to enhance BAT activity and WAT browning, increasing UCP1 expression in a dose-dependent manner [102]. In a pioneering human study, Cypess et al. showed that a dose (200 mg/day, orally) of mirabegron leads to higher BAT metabolic activity and further increased energy expenditure in healthy lean men [103]. In addition, in young healthy women, mirabegron acutely increased BAT activity, also increasing their metabolic rate and energy expenditure [104]. In the same study, the effects of mirabegron were more prominent in subjects with low BAT amount at baseline after 4 weeks of therapy, highly increasing BAT mass and ameliorating the lipid profile, insulin sensitivity, and secretion in the subjects, with no changes in body weight and composition [104]. An increase in skin temperature and improved glucose homeostasis were also reported after mirabegron administration in humans [105]. Further studies in obese insulin-resistant patients demonstrated that chronic mirabegron treatment induces UCP1 expression in sWAT and thermogenic PGC1α marker in skeletal muscle, while also increasing the number of oxidative fibers [19]. However, no effects on body weight and/or fat mass reduction were found. Although mirabegron has great thermogenic potential in anti-obesity therapies, the lack of significant weight-lowering effects and the cardiovascular side effects reported in diverse studies may limit its broad use in humans. Further long-term human studies are also needed to shed light on the applicability of mirabegron in limiting obesity.

**Thyroid hormone receptor (THR) agonists**, such as levothyroxine (C_15_H_11_I_4_NO_4_) and liothyronine (C_15_H_12_I_3_NO_4_), have also been investigated for their roles in metabolic regulation. While most evidence is derived from observational data linking thyroid disorders with weight changes, hyperthyroid patients and subjects under TH replacement therapy reported increased energy expenditure and body temperature, also showing reduced body weight and cholesterol levels associated with enhanced BAT activation and increased muscle glucose uptake [106]. Triiodothyronine (T3) has shown browning potential by promoting a brown-like adipocyte phenotype in human adipose-derived stem cells, increasing UCP1 expression, mitochondrial biogenesis, and oxidative metabolism [107]. In rodents, T3 also stimulates thermogenesis in BAT, WAT, and skeletal muscle, regulating fiber composition and SERCA isoform expression through AMPK and UCP1 activation after THR β (THRB) activation [106]. Selective THRB agonists like Resmetirom (C_17_H_12_Cl_2_N_6_O_4_), recently approved for nonalcoholic steatohepatitis, improve cholesterol and triglyceride levels and present strong anti-obesity potential [108], though human studies remain limited.

**Farnesoid X receptor (FXR) agonists**, known for regulating bile acid and lipid metabolism, also show promising thermogenic effects. In obese mice, fexaramine (C_32_H_36_N_2_O_3_) enhanced thermogenic gene expression in BAT and WAT, promoting mitochondriogenesis and fat oxidation [109]. Moreover, farnesol (C_15_H_26_O) treatment also activates the AMPK/PGC1α/UCP1 thermogenic pathway in both mice and human adipocytes [110], and oral supplementation of chenodeoxycholic acid (C_24_H_40_O_4_), a bile acid that stimulates the farnesoid X receptor, increases basal energy expenditure, promoting BAT activity and cold-induced thermogenesis in women [111]. While some FXR agonists are under clinical investigation for metabolic syndrome, their direct role in thermogenesis and WAT browning in humans is still unclear.

**Growth hormone (GH)** deficiency, such as somatotropin (C_990_H_1529_N_263_O_299_S_7_) deficiency, is associated with increased body fat and altered distribution. GH replacement therapy in humans restores basal metabolic rate, reduces visceral fat and waist circumference, and improves lipid profiles, glucose tolerance, and insulin sensitivity, recovering body composition [112] and highlighting GH’s link with central obesity and diabetes. Tesamorelin (C_221_H_366_N_72_O_67_S), a synthetic analogue of GH-releasing factor, also enhanced phosphocreatine recovery post-exercise in obese subjects [113], though no direct effects on BAT or WAT-mediated NST have been confirmed in humans. In rodents, GH therapy has controversial effects on BAT activity and WAT browning (as reviewed in [114]).

**Glucagon-like peptide 1 (GLP-1) receptor agonists** have emerged as pivotal tools in anti-diabesity therapy due to their ability to enhance insulin secretion, inhibit glucagon release, reduce appetite, and promote sustained weight loss [101]. Despite the absence of GLP-1 receptor expression in WAT, in vitro studies showed that GLP-1 analogs like exendin-4 (C_184_H_282_N_50_O_60_S) promote adipocyte browning with an increase in thermogenic gene expression via AMPK/SIRT1/PGC1α/UCP1 pathway stimulation [115]. In rodents, liraglutide (C_172_H_265_N_43_O_51_), a long-acting GLP-1 receptor agonist, stimulated BAT thermogenesis and promoted WAT browning, resulting in reduced body weight and food intake [116]. In humans, liraglutide effects were also confirmed, showing significant weight loss, improved metabolic markers, and increased energy expenditure in obese and diabetic patients [116]. Once-weekly semaglutide (C_187_H_291_N_45_O_59_), a newer and oral GLP-1 analog, demonstrated even greater weight loss and appetite suppression effects, reducing visceral fat mass and enhancing metabolic control in humans [117], making it the most effective GLP-1 analog to date. The thermogenic role of GLP-1 receptor agonists in humans remains scarce; however, enhanced BAT activity and WAT browning after semaglutide treatment have been reported in obese mice [118]. Tirzepatide (C_225_H_348_N_48_O_68_), a dual glucose-dependent insulinotropic polypeptide receptor (GIPR) and GLP-1 receptor agonist, has recently gained importance for its synergistic effects on energy metabolism. GIPR stimulation in human adipose-derived stem cells promotes browning, UCP1 upregulation, and lipid metabolism via PKA signaling [119]. Clinical studies, like SURMOUNT trials, show that tirzepatide has greater anti-obesity effects than semaglutide, promoting weight and fat mass reduction, appetite suppression, energy expenditure, insulin sensitivity, and glycemic parameters in obese [120] and diabetic patients [121]. Although thermogenesis was not evaluated in SURMOUNT studies, an ongoing clinical trial (NCT04081337) will elucidate tirzepatide’s role in energy expenditure and NST. Tirzepatide was recently approved not just for T2DM treatment but also for the treatment of obesity and overweight, particularly in people with related metabolic conditions [120]. Additionally, dual GLP-1/glucagon agonists such as oxyntomodulin (C_192_H_295_N_59_O_60_S) [122] and mazdutide (C_207_H_317_N_45_O_65_) have shown encouraging outcomes in promoting weight loss, reducing body mass index, waist circumference, and food intake, increasing energy expenditure, and improving glycemic control in obese and/or overweight subjects [123]. Retatrutide (C_221_H_342_N_46_O_68_), a triple GLP-1/GIP/glucagon receptor agonist, is currently under study to find its potential in anti-diabesity therapies; however, clinical relevance is not well established. In humans, retatrutide reduces body weight while improving glucose homeostasis, insulin sensitivity, and lipid profile [124]. Despite the promising clinical effects of these agonists, their precise impact on human thermogenesis, including BAT activation and WAT browning, remains insufficiently characterized. Future studies measuring energy expenditure, thermogenic gene expression, and adipose tissue remodeling are essential to fully define their roles in thermogenesis-based anti-diabesity therapies.

**Sodium-glucose cotransporter 2 (SGLT2) inhibitors** are emerging as promising anti-diabesity drugs due to their anti-hyperglycemic and body weight regulatory effects. In obese rodents, these drugs have demonstrated the ability to limit weight gain, enhance energy expenditure, and promote thermogenesis via upregulation of UCP1 in both BAT and WAT and increased muscle fat oxidation by activating the AMPK/SIRT1 signaling axis [125]. In humans, dapagliflozin (C_21_H_25_ClO_6_) reduced body weight, waist circumference, insulin resistance, and fat mass and improved glycemia, insulin sensitivity, respiratory exchange ratio, and the lipid profile of diabetic patients [126]. Empagliflozin (C_23_H_27_ClO_7_) and canagliflozin (C_24_H_25_FO_5_S) similarly reduced body weight and fat mass, and both restored glycemic parameters in diabetic subjects [127]. While these agents were designed to flush out glucose, they may also ignite dormant thermogenic circuits, offering a twofold strike against diabesity. More mechanistic human studies are needed to uncover the full thermogenic potential of SGLT2 inhibitors and validate their role in targeting energy balance through adipose tissue and skeletal muscle NST.

### 7.2. Melatonin

**Melatonin** (N-acetyl-5-methoxytryptamine, C_13_H_16_N_2_O_2_) is a highly conserved hormone present across the plant and animal kingdoms. In mammals, melatonin is predominantly synthesized at night by the pineal gland, but also in many other organs [128], and is widely exogenously administered in humans in Europe and the USA both as a drug and as a supplement [129]. Melatonin levels naturally decline with age, shift work, exposure to artificial light, and in metabolic diseases such as diabesity [130]. Beyond its well-established antioxidant and anti-inflammatory properties, melatonin is gaining importance for its metabolic and thermogenic roles [128].

In humans, however, data from several small-scale (<100 participants) studies on the use of melatonin in patients with metabolic syndrome or obesity are conflicting and inconclusive, ranging from a modest effect to no effect, depending on the protocol applied (melatonin dosage and duration) (reviewed in [129]). In this scenario, a systematic review and meta-analysis of 20 human studies proved that melatonin significantly reduced body weight, and in more than 50% of the studies, decreased body mass index, weight gain, or waist circumference in men and women, showing better outcomes with higher melatonin doses (8 mg/day) and longer treatment durations (48 weeks) [131]. A recent systematic review and dose–response meta-analysis of 28 randomized controlled clinical trials also exhibited that melatonin supplementation reduces hip circumference in both sexes and waist circumference in interventions longer than 8 weeks in overweight and/or diabetic subjects, also improving the lipid profile and reducing fat accumulation. Body weight in diabetic patients and body mass index in obese individuals were also lower after melatonin treatment compared to placebo [132]. However, numerous clinical trials show that melatonin has no effect on body weight loss in obese and/or diabetic subjects, results not comparable to those obtained in rodents, perhaps because of the great genetic variability of melatonin receptors in humans, especially the membrane melatonin receptor 2 (MT2) [129].

A substantial amount of promising data regarding the potential role of melatonin-based therapy in the prevention of obesity and its related metabolic disorders has been reported in preclinical studies. Various mechanisms have been proposed to explain the beneficial effects of melatonin on body weight and metabolism, and several receptors have been proposed, including the membrane receptors MT1 and MT2, the main receptors studied regulating melatonin’s effects on obesity [133]. (i) Since increased core body temperature and no changes in food intake have been found, and locomotor activity data in rodents are inconsistent, one mechanism may involve the increase in energy expenditure by NST activating BAT and/or WAT browning through UCP1 upregulation [133], and/or skeletal muscle NST through SERCA/SLN uncoupling and CaMKII/AMPK/PGC1α pathway activation [134]. (ii) Another mechanism is through its well-known chronobiotic effects in both humans and rodents, participating in the control of metabolism and energy balance of the organism over 24 h cycles, increasing insulin sensitivity and the energy intake use and/or storage during the active phase (day), and reducing it during the rest/sleep phase (night), accentuating adipose tissue lipolysis [130,135]. (iii) Also, melatonin improves diabesity through its antioxidant and anti-inflammatory properties and its potential to maintain mitochondrial homeostasis and function in adipose tissue and skeletal muscle [133,136]. (iv) Recently, studies showed that melatonin is also produced by microbiota, and exogenous supplementation can improve microbiota dysbiosis, alleviating weight gain and fat accumulation, reducing low-grade inflammation, and promoting lipid metabolism and energy expenditure [133]. In the following section, we will focus on the effect of melatonin on the recovery of mitochondrial function in obesity.

Our previous studies showed that chronic melatonin treatment reduced body weight gain in a preclinical animal model of diabesity, assembling human obesity-related type 2 diabetes, browned subcutaneous fat, increased thermogenic BAT function and mass, enhanced skeletal muscle NST and oxidative fiber phenotype, and improved lipid profile, glucose homeostasis, low-grade inflammation, and mitochondrial biogenesis and functionality [134,137,138,139,140,141,142,143,144]. These changes took place in the absence of changes in locomotor activity or feeding patterns, suggesting a thermogenic effect. These outstanding findings encourage further research on the translation of these findings on melatonin effects to humans. The beneficial effect of melatonin and its analogs against a wide range of diseases prompted many decades of studies to evaluate the safety aspect of this substance. A substantial number of both human and animal studies reported that short-term use of melatonin possesses no adverse effects, even in high doses, whereas long-term melatonin treatment in humans causes only mild adverse effects such as dizziness, headache, nausea, and sleepiness. However, due to a lack of studies, it is difficult to exclude any possible adverse effects in pregnant and breastfeeding women, as well as in children and adolescents. In experimental animal studies, the lethal dose has not been estimated; even high doses up to 800 mg/kg are without any acute toxic effects. Also, studies in healthy subjects indicate that a dose of 10 mg for 28 days shows no toxicity [145].

Melatonin works via multiple means to limit oxidative stress: (i) One mechanism is by acting as a direct scavenger to detoxify free oxygen and nitrogen species and stimulating the expression and activity of some antioxidant enzymes (e.g., NRF1/2, glutathione, GPx, SOD2, and SIRT1/3) [133,146]. (ii) Another mechanism is by stabilizing mitochondrial membrane integrity, thereby preventing the opening of the mitochondrial permeability transition pore (mPTP) and cytochrome C release into the cytosol, inhibiting pro-apoptotic pathways, regulating mitochondrial dynamics and autophagy, and promoting mitochondrial quality [136,147,148]. (iii) Also, it works by improving electron transport chain activity and mitochondrial function (increasing ATP production and the activities of respiratory chain Complex I-IV) [147,148,149]. (iv) In addition, melatonin can also modify the metabolic fuel used, regulating energy balance and increasing lipolysis and metabolism switching into a fat oxidative one, therefore reducing body intra-abdominal fat [133,142,150,151]. (v) Finally, melatonin also shows cell protective effects, preventing ER-stress mediated apoptosis [152]. Several of these melatonin effects have already been demonstrated in thermogenic tissues such as BAT [137], WAT [144,153,154], and skeletal muscle [142,150,155,156], being additional mechanisms to NST that regulate the energy balance and limit diabesity. Furthermore, melatonin positively affects muscle metabolism, enhancing insulin sensitivity and glucose homeostasis; preserving its structure; increasing myofiber size, mitochondrial function, membrane integrity, and dynamics; promoting myofiber differentiation; reducing oxidative stress, inflammation, and apoptosis in both human and preclinical models of diabesity; and preventing muscle loss and atrophy known as sarcopenia [133].

Based on all of melatonin’s aforementioned effects concerning its beneficial impact on body weight, the potential thermogenic effect, the safety aspect, and the powerful antioxidant properties, melatonin should be considered as a promising therapeutic option in addition to the drugs mentioned above for obesity management by activating thermogenic mechanisms (Table 2 and Table 3 and Figure 5).

**Table 2:** Summary of the physiological conditions, bioactive agents, and drugs stimulating non-shivering thermogenesis (NST) in rodents with their anti-diabesity effect, thermogenic mechanism, and molecular pathway involved.

Stimuli		Anti-Diabesity Effect	Mechanism	Molecular Pathway	References
Physiological conditions	Cold exposure	↑ Energy expenditure ↑ Insulin sensitivity	↑ BAT activity and recruitment ↑ BAT & WAT UCP1 expression ↑ WAT browning & angiogenesiss ↑ SKM SLN expression ↓ SKM SERCA activity ↑ SKM mitochondrial function	TRPs/β3-ARs/PPARγ/PGC1α	[14,15,17,20]
	Physical exercise	↓ Insulin resistance ↑ Glucose sensitivity ↑ Lipid metabolism ↓ Fat mass	↑ BAT activity and recruitment ↑ WAT browning & mitochondrial function ↑ WAT UCP1 expression ↓ vWAT adiposity ↑ SKM UCP3, SERCA1 & SLN expression ↑ SKM mitochondrial biogenesis and function	PGC1α/FNDC5/Irisin	[26,28,29,30]
	Fasting	↓ Body weight ↑ Energy expenditure	↑ BAT activation ↑ BAT & SKM energy expenditure ↑ WAT browning ↑ BAT & WAT UCP1 expression	SCFA (Gut microbiota)/CD36/ β3-ARs/SIRT6/PGC1α	[31,33,34,35,37,44]
Bioactive ingredients	Capsaicin & Capsinoids 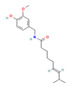 (Pepper)	↓ Weight gain ↑ Energy expenditure ↓ Fat accumulation Improved glucose levels	↑ BAT activity and recruitment ↑ WAT browning ↑ BAT & WAT UCP1 expression ↑ SKM UCP2/3, SERCA1/2 & RyR1/2 expression ↑ SKM SERCA activity & ATP hydrolysis	TRPV1/β3-ARs/CaMKII/AMPK/ SIRT1/PGC1α	[46,48]
	6-Paradol, 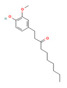 Gingerol 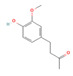 & Zingerone 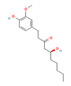 (Ginger)	↓ Body weight ↓ Fat mass ↑ Energy expenditure	↑ BAT activity & function ↑ vWAT, sWAT & white adipocytes (in vitro) browning ↑ BAT & WAT UCP1 expression ↓ WAT adipogenesis & ↑ lipolysis ↑ SKM mitochondrial biogenesis	TRPV1/β3-ARs/AMPK/SIRT1/ PPARα/PGC1α	[51,52,53,54]
	Cinnamaldehyde 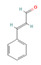 & Cinnamic acid 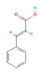 (Cinnamon)	↓ Body weight ↑ Energy expenditure	↑ Body temperature ↑ BAT activity & brown adipocytes (in vitro) function ↑ BAT mitochondrial ATP production ↑ vWAT, sWAT & white adipocytes (in vitro) browning ↑ BAT & WAT UCP1 expression ↓ Adipocytes size & lipogenesis (in vitro)	TRPA1/β3-ARs/FGF21/AMPK/ PPARγ/PGC1α	[55,56,57,59]
	Curcumin 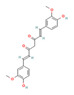 (Turmeric)	↓ Body weight ↑ Energy expenditure ↑ Insulin sensitivity	↑ BAT activation ↑ sWAT & white adipocytes (in vitro) browning ↑ BAT & sWAT UCP1 expression ↑ Muscle cells mitochondrial function & energy expenditure (in vitro) ↑ SKM SERCA1 expression ATP hydrolysis & fiber type composition modulation	FNDC5/Irisin/β3-ARs/AMPK/ PPARγ/PGC1α	[61,62,63]
	Allicin 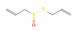 (Garlic)	↓ Weight gain ↑ Energy expenditure Improved glucose homeostasis	↑ BAT activation & fat oxidation ↑ WAT & white adipocytes (in vitro) browning ↑ BAT & sWAT UCP1 expression ↑ SKM UCP3 expression ↑ Lipolysis	β3-ARs/AMPK/SIRTs/ PPARα/PGC1α	[65]
	Quercetin 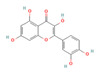 (Onion)	↑ Weight loss ↑ Energy expenditure ↓ Fat accumulation Improved glucose homeostasis	↑ BAT mass & function ↑ sWAT & white adipocytes (in vitro) browning ↑ BAT & WAT UCP1 expression ↑ Fat oxidation & ↓ adipogenesis ↑ SKM mitochondrial function & biogenesis SKM SERCA1/2 activity & function modulation SKM SERCAs conformational regulation ↑ SKM glucose uptake	β3-ARs/FGF21/PKA/AMPK/ SIRT1/PPARα/γ/PGC1α	[67,68,69]
	Caffeine 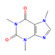 (Coffee)	↑ Weight loss ↑ Energy expenditure	↑ White adipocytes browning & UCP1 expression (in vitro) ↑ BAT UCP1 expression ↑ SKM UCP3 expression SKM RyR agonist & SERCA activity uncoupler ↑ Mitochondriogenesis	RyR/SERCA/PPARγ/PGC1α	[70,71,72]
	Catechins 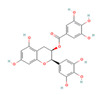 & Theaflavins 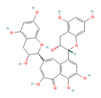 (Tea)	↓ Body weight ↑ Energy expenditure	↑ BAT activity & function ↑ BAT & WAT UCP1 expression ↑ WAT & white adipocytes (in vitro) browning =/↑ SKM UCP3 expression =SKM FNDC5/Irisin & SLN expression	TRPA/V1/β3-ARs/BMP7/FGF21/ Adiponectin/AMPK/SIRT1/ PPARγ/PGC1α	[73,74,76]
	Flavan-3-ols 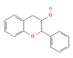 (Cocoa)	↑ Energy expenditure	↑ BAT activation & UCP1 expression ↑ SKM UCP3 expression ↑ Mitochondriogenesis	β3-ARs/AMPK/PGC1α	[77,78]
	Berberine 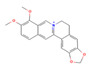 (Chinese goldthread, goldenseal)	↓ Body weight ↑ Energy expenditure Improved glucose homeostasis	↑ BAT activity ↑ sWAT & white adipocytes (in vitro) browning ↑ Brown adipocytes differentiation (in vitro) ↑ BAT & WAT UCP1 expression ↑ Mitochondrial biogenesis & function	AMPK/SIRT1/PPARγ/PGC1α	[79,80]
	DHA 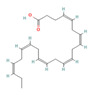 & EPA 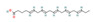 (Oily fish)	↓ Weight gain ↑ Energy expenditure Improved glucose metabolism	↑ BAT mass & activity ↑ WAT browning ↑ BAT, sWAT & brown and white adipocytes (in vitro) UCP1 expression ↑ Mitochondrial function & biogenesis ↑ Fat & glucose oxidation ↑ SKM SERCA activity uncoupling ↑ SKM SERCA2b & SLN expression ↑ SKM development	TRPV1/β3-ARs/FGF21/ Irisin/AMPK/SIRT1/PGC1α	[81,82,83,85,86]
	Eriocitrin 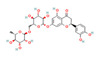 (Lemon)	↑ Energy expenditure ↑ Insulin sensitivity	↑ BAT UCP1 expression ↑ SKM UCP3, SERCA1/2 & SLN expression ↑ Fat oxidation	?	[87]
	Menthol 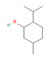 (Mint)	↑ Weight loss ↑ Energy expenditure Improved glucose metabolism	↑ BAT activity & fat oxidation ↑ WAT & white adipocyte (in vitro) browning ↑ BAT, WAT & brown and white adipocytes (in vitro) UCP1 expression ↑ Mitochondrial activity & metabolic rate ↑ SKM energy expenditure ↑ Skin temperature	TRPM8/β3-ARs/FGF21/ Calcium/PKA/AMPK/PGC1α	[88,89]
	Thymol 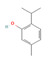 (Thyme)	?	↑ White adipocytes browning & UCP1 expression (in vitro) ↑ Mitochondriogenesis (in vitro)	β3-ARs/PKA/AMPK	[91]
	Resveratrol, 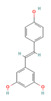 Proanthocyanidin 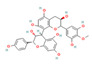 & Anthocyanin 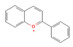 (Grapes & Berries)	↓ Body weight ↑ Energy expenditure ↓ Fat mass ↓ Insulin resistance Improved glycemic and lipid profile	↑ BAT activity & recruitment ↑ WAT & white adipocytes (in vitro) browning ↑ BAT, WAT & brown and white adipocytes (in vitro) UCP1 expression ↑ Fat oxidation & ↓ vWAT adipogenesis ↑ SKM UCP3 expression ↑ SKM glucose uptake ↑ SKM, BAT, WAT, brown and white adipocytes (in vitro) mitochondrial dynamic & function	β3-ARs/BMP7/FNDC5/Irisin/ERα/ AMPK/SIRT1/3/PPARα/γ/PGC1α	[92,93,95,96,97,98,99,100]
Drugs	β3-AR agonists Mirabegron ** 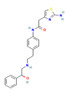 **	↑ Weight loss ↑ Energy expenditure ↑ Insulin sensitivity and secretion Improved lipid profile and glucose homeostasis	↑ BAT mass & activity ↑ WAT & white adipocytes (in vitro) browning & UCP1 expression	β3-ARs/PGC1α	[102]
	THR agonist Levothyroxine 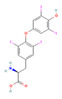 Liothyronine 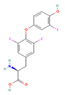 Resmetirom 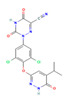	↓ Body weight ↑ Energy expenditure Improved lipid profile	↑ BAT activity & function ↑ WAT browning ↑ BAT & WAT UCP1 expression	THRB/AMPK	[106]
	FXR agonist Fexaramine 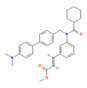 Farnesol 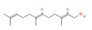 CDCA 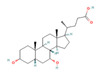	↑ Energy expenditure	↑ BAT mitochondriogenesis & fat oxidation ↑ WAT browning ↑ BAT, WAT & white and brown adipocytes (in vitro) UCP1 expression Adipogenesis modulation	FXR/AMPK/PPARγ/PGC1α	[109,110]
	Growth Hormone Tesamorelin 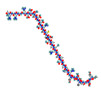	↓ Fat mass Improved lipid profile, glucose tolerance and insulin sensitivity	=/↑ BAT activity =/↑ WAT browning	GHR	[114]
	GLP1R agonists Liraglutide 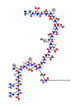 Semaglutide 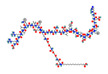 Exedin-4 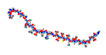 GLP1R/GIPR dual agonist Tirzepatide 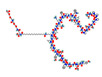 GLP1R/GR dual agonist Oxyntomodulin 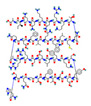 Mazdutide 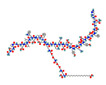 GLP1R/GR/GIPR triple agonist Retatrutide 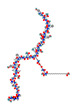	↑ Weight loss ↑ Energy expenditure ↓ Food intake ↓ Fat mass Improved insulin sensitivity and secretion, and glycemic control	↑ BAT activity & function ↑ WAT & white adipocytes (in vitro) browning ↑ BAT, WAT & white adipocytes (in vitro) UCP1 expression ↑ SKM mitochondrial function & thermogenic genes expression	GLP1R/PKA/AMPK/SIRT1/PGC1α	[115,116,118]
	SGLT2 Inhibitors Dapagliflozin 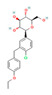 Empagliflozin 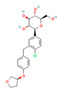 Canagliflozin 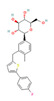	↓ Body weight ↑ Energy expenditure ↓ Fat mass Improved lipid profile, glucose homeostasis and insulin sensitivity	↑ BAT activity & WAT browning ↑ BAT & WAT UCP1 expression ↑ SKM fat oxidation	SGLT2/AMPK/SIRT1	[125]
	Melatonin 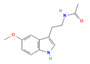	↑ Energy expenditure ↓ Body weight ↓ Visceral fat mass Improved lipid profile, glucose homeostasis and insulin sensitivity	↑ Body temperature ↑ BAT activity, mass & function ↑ sWAT browning ↑ BAT & WAT UCP1 expression ↑ SKM SERCA activity & expression ↑ SKM SLN expression ↑ BAT, WAT & SKM mitochondrial quality, function and biogenesis ↑ Mitochondrial membrane integrity & dynamics ↓ Organellar stress & apoptosis ↓ Fat accumulation, oxidative stress & low-grade inflammation ↑ Lipid metabolism Improved SKM fiber composition, microbiota dysbiosis, metabolism control and plasticity & energy balance over 24 h	MT1/2/PDK1/Akt/CaMKII/AMPK/ SIRT1/3/PGC1α/NRF1/2/SOD2	[134,137,138,139,140,141,142,143,144,150,151,153,154,155,156]

↑, increase in; ↓, decrease in; =, no changes in; Akt, protein kinase B; AMPK, 5′ adenosine monophosphate-activated protein kinase; ATP, adenosine triphosphate; β3-AR, β3 adrenergic receptor; BAT, brown adipose tissue; BMP7, bone morphogenetic protein 7; CaMKII, Ca^2+^/calmodulin-dependent protein kinase II; CD36, cluster of differentiation 36; CDCA, Chenodeoxycholic acid; DHA, docosahexaenoic acid; EPA, eicosapentaenoic acid; ERα, estrogen receptor α; FGF21, fibroblast growth factor 21; FNDC5, fibronectin type III domain-containing protein 5; FXR, farnesoid X receptor; GHR, growth hormone receptor; GIPR, glucose-dependent insulinotropic polypeptide receptor; GLP1R, glucagon-like peptide-1 receptor; GR, glucagon receptor; MT1/2, melatonin receptor 1/2; NRF1/2, nuclear respiratory factor 1/2; PDK1, pyruvate dehydrogenase kinase 1; PGC1α, peroxisome proliferator-activated receptor gamma co-activator 1α; PKA, protein kinase A; PPARα/γ, peroxisome proliferator-activated receptor α/γ; RyR, ryanodine receptor; SCFA, short-chain fatty acids; SERCA, sarcoplasmic/endoplasmic reticulum Ca^2+^-ATPase; SGLT2, sodium-glucose linked transporter 2; SIRT1/3/6, sirtuin 1/3/6; SKM, skeletal muscle; SLN, sarcolipin; SOD2, superoxide dismutase 2; sWAT, subcutaneous white adipose tissue; TH, thyroid hormone; THR, thyroid hormone receptor; THRB, thyroid hormone receptor β; TRP, transient receptor potential; TRPA1, transient receptor potential A1; TRPM8, transient receptor potential cation channel melastatin 8; TRPV1, transient receptor potential vanilloid 1; UCP1, uncoupling protein 1; UCP2, uncoupling protein 2; UCP3, uncoupling protein 3; vWAT, visceral white adipose tissue; WAT, white adipose tissue.

**Table 3:** Summary of human studies and clinical trials on non-shivering thermogenesis (NST) stimulation through physiological conditions, bioactive agents, and thermogenic drugs in diabesity.

Stimuli		Type of Study	Anti-Diabesity Effect	Mechanism	References
Physiological conditions	Cold exposure	Clinical Trial	↑ Energy expenditure ↑ Insulin sensitivity	↑ BAT activity and recruitment ↑ sWAT UCP1 expression & mitochondrial function ↑ SKM GLUT4 expression	[18,19,21]
	Physical exercise	Observational Human study	↓ Insulin resistance ↑ Glucose sensitivity ↑ Lipid metabolism ↓ Visceral fat mass	↑ BAT activation ↑ WAT browning ↑ BAT & WAT UCP1 expression ↑ SKM UCP3 expression	[24,25,27]
	Fasting	Clinical Trial	↓ Body weight ↑ Energy expenditure	= sWAT UCP1 expression	[32,33]
		In vivo Human study	Improved metabolic health	↑ SKM UCP3 expression	[45]
Bioactive ingredients	Capsaicin & Capsinoids 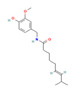 (Pepper)	Clinical Trial	↓ Weight gain ↑ Energy expenditure ↓ Abdominal fat accumulation Restored glucose levels	↑ BAT activity ↑ Fat oxidation	[47]
	6-Paradol 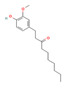 (Grain of Paradise)	Clinical Trial	↓ Body weight ↓ Visceral fat mass ↑ Energy expenditure	↑ BAT activation and recruitment	[49,50]
	Cinnamaldehyde 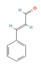 & Cinnamic acid 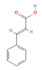 (Cinnamon)	In vitro Human study	↑ Metabolic response	↑ Adipocytes browning & UCP1 expression ↑ Fat oxidation	[57]
		Clinical Trial	↓ Body weight ↑ Energy expenditure	↑ Facial skin temperature	[58]
	Curcumin 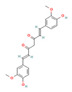 (Turmeric)	Clinical Trial	↓ Body weight ↓ Waist/hip circumference ↑ Energy expenditure ↑ Insulin sensitivity	↓ Fat mass & anthropometric measurements	[60]
	Allicin 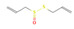 (Garlic)	In vitro Human study	↓ Weight gain ↑ Energy expenditure Improved glucose tolerance	↑ White adipocytes browning & UCP1 expression	[66]
	Caffeine 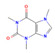 (Coffee)	In vitro & in vivo Human study	↑ Weight loss ↑ Energy expenditure	↑ BAT activity & function ↑ Adipocytes browning & UCP1 expression = SKM UCP3 expression	[71]
	Catechins 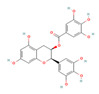 (Tea)	Clinical Trial	↓ Body weight ↑ Energy expenditure	↑ BAT activation and recruitment	[75]
	Berberine 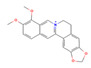 (Chinese goldthread, goldenseal)	In vitro & in vivo Human study	↓ Body weight ↑ Energy expenditure Improved glucose homeostasis Restored metabolic health	↑ BAT mass & function ↑ BAT recruitment & brown adipogenesis	[80]
	EPA 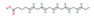 (Oily fish)	In vitro & in vivo Human study	↓ Weight gain ↑ Energy expenditure Improved glucose metabolism	↑ Subcutaneous white adipocytes browning = SKM SERCA activity & function	[84]
	Menthol 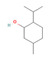 (Mint)	Clinical Trial	↑ Weight loss ↑ Energy expenditure Improved glucose metabolism	↑ Skin temperature & metabolic rate	[90]
	Resveratrol 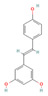 (Grapes & Berries)	In vitro & in vivo Human study	↓ Body weight ↑ Energy expenditure ↓ Visceral fat mass ↓ Insulin resistance Improved glycemic and lipid profile	↑ sWAT & white adipocytes browning ↑ sWAT & white adipocytes UCP1 expression ↑ SERCA activity and expression & calcium modulation	[93,94]
Drugs	β3-AR agonists Mirabegron ** 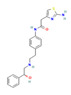 **	Clinical Trial	↑ Weight loss ↑ Energy expenditure ↑ Insulin sensitivity and secretion Improved lipid profile and glucose homeostasis	↑ BAT mass & activity ↑ BAT energy expenditure & metabolic rate ↑ sWAT browning & UCP1 expression ↑ SKM PGC1α expression ↑ SKM oxidative fibers ↑ Supraclavicular skin temperature	[19,103,104,105]
	THR agonist Levothyroxine 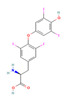 Liothyronine 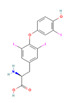 Resmetirom 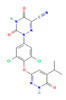	In vitro & in vivo Human study	Improved metabolism homeostasis	↑ BAT activity & function ↑ Adipocytes browning & UCP1 expression ↑ SKM glucose uptake & function ↑ SKM SERCA activity and expression SKM fiber composition modulation ↑ Mitochondrial biogenesis & oxidative metabolism	[106,107]
		Clinical Trial	↓ Body weight ↑ Energy expenditure Improved lipid profile	↑ Body temperature	[108]
	FXR agonist Farnesol 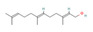 CDCA 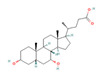	In vitro Human study	↓ Fat accumulation ↑ Metabolic response	↑ Adipocytes browning & UCP1 expression	[110]
		Clinical Trial	↑ Energy expenditure	↑ BAT activity & function	[111]
	Growth Hormone Tesamorelin 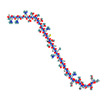	Clinical Trial	↑ Energy expenditure ↓ BMI, waist circumference ↓ Visceral fat mass Improved lipid profile, glucose tolerance and insulin sensitivity	↑ SKM mitochondrial function & phosphocreatine recovery	[112,113]
	GLP1R agonists Liraglutide 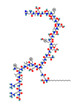 Semaglutide 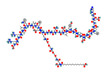 GLP1R/GIPR dual agonist Tirzepatide 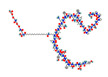 GLP1R/GR dual agonist Oxyntomodulin 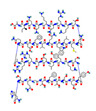 Mazdutide 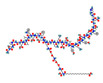 GLP1R/GR/GIPR triple agonist Retatrutide 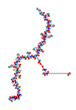	In vitro Human study	↓ Fat accumulation	↑ Adipocytes browning ↑ Lipid metabolism	[119]
		Clinical Trial	↑ Weight loss ↑ Energy expenditure ↓ BMI, waist circumference, and food intake ↓ Visceral fat mass Improved insulin sensitivity and secretion, and glycemic control	↑ Supraclavicular & neck BAT activity ↑ Lipid metabolism	[116,117,120,121,122,123,124]
	SGLT2 Inhibitors Dapagliflozin 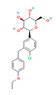 Empagliflozin 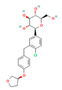 Canagliflozin 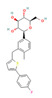	Clinical Trial	↓ Body weight ↑ Energy expenditure ↓ Waist circumference, waist-to-height ratio ↓ Fat mass Improved lipid profile, glucose homeostasis and insulin sensitivity	↑ SKM fat oxidative metabolic rate Improved respiratory exchange ratio	[126,127]
	Melatonin 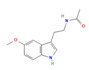	Clinical Trial	↑ Energy expenditure ↓/=/↑ Body weight ↓/= BMI & waist and hip circumference ↓ Visceral fat mass Improved lipid profile, glucose homeostasis and insulin sensitivity	↑ BAT activity, mass & function ↓ Oxidative stress	[131,132,145]

↑, increase in; ↓, decrease in; =, no changes in; β3-AR, β3 adrenergic receptor; BAT, brown adipose tissue; BMI, body mass index; CDCA, Chenodeoxycholic acid; EPA, eicosapentaenoic acid; FXR, farnesoid X receptor; GIPR, glucose-dependent insulinotropic polypeptide receptor; GLP1R, glucagon-like peptide-1 receptor; GLUT4, glucose transporter type 4; GR, glucagon receptor; PGC1α, peroxisome proliferator-activated receptor gamma co-activator 1α; SERCA, sarcoplasmic/endoplasmic reticulum Ca^2+^-ATPase; SGLT2, sodium-glucose linked transporter 2; SKM, skeletal muscle; sWAT, subcutaneous white adipose tissue; THR, thyroid hormone receptor; UCP1, uncoupling protein 1; UCP3, uncoupling protein 3; WAT, white adipose tissue.

## 8. Limitations of the Study

This review has several inherent limitations that should be considered when interpreting its results. First, the literature search was conducted using only MEDLINE via PubMed and Web of Science and included only peer-reviewed original articles, systematic reviews, and narrative reviews published in English. Although no date restrictions were applied, the literature search was not exhaustive, and language and database limitations may have excluded relevant studies published in other languages or indexed elsewhere. In addition, the quality of the studies included varied, and many did not use standardized methods to evaluate the outcomes of interest. Furthermore, the review does not include a formal assessment of the quality of the studies included. In addition, the predominance of preclinical studies in certain sections also represents a limitation in terms of the applicability and translationality of these thermogenic agents. More human studies are needed in this field as the overall clinical evidence remains scarce, especially with regard to well-designed randomized controlled trials.

This review provides a comprehensive and detailed overview of the impact of thermogenesis in the fight against diabesity, while also serving as an update on the state of the art in this field of research. However, among clinical studies, which tend to have a more robust methodology, many of them are observational and mainly measure indirect thermogenesis through anthropometric outcomes, such as weight loss or fat reduction. Direct measurements of NST through thermogenic markers are rarely evaluated, with results based on indirect measurements. Therefore, the heterogeneity in the assessment criteria and methodologies used introduces a bias that could affect the comparability and validity of the conclusions, and it is important for the scientific community to adopt standard methodologies in future clinical studies on thermogenesis.

The narrative nature of this review offers a broad and comprehensive perspective on a complex topic, but as a result, the level of evidence presented is less definitive than that of meta-analyses focused on more specific research questions where quantitative analysis is possible, with this also being a limitation of the current review.

Therefore, future research should address these limitations by conducting randomized clinical trials with sufficient statistical power, standardized protocols, direct measures of NST, and incorporating diverse populations that include different genders, ages, and metabolic states. In addition, exploring multi-target interventions that combine physiological stimuli, bioactive compounds from the diet, and pharmacological treatments could provide more effective strategies against diabesity, a question that remains unexplored.

## 9. Conclusions and Future Perspectives

After the discovery that brown and beige fat cells exist in human adults and contribute to energy expenditure, white-fat browning and BAT activation/recruitment, together with skeletal muscle SERCA/SLN-mediated NST, are regarded as a promising alternative strategy to treat obesity and related consequences. In this context, the main challenge now is to identify molecules that will directly target brown and beige fat cells and SLN expression in muscle and produce the intended metabolic benefits in humans, with minimal side effects. In recent decades, tremendous efforts have been made towards testing the potential of many treatments and physiological manipulations to induce WAT browning or BAT activity/recruitment.

At first, food-sourced bioactive compounds are gaining high interest in obesity research, and extensive studies are being performed towards their possible thermogenic effect and metabolic benefit. Current evidence from animal models, cell culture models, and limited human studies highlights the promising potential of physiological stimuli, bioactive compounds, and pharmacological agents to activate the thermogenic program as a therapeutic strategy against diabesity. However, rigorous, well-designed randomized controlled trials in humans are urgently needed to validate these findings and assess their translational value in the current clinical practice. It is essential that these clinical trials incorporate comprehensive assessments of thermogenic activity using direct molecular markers, such as UCP1 and SLN expression, mitochondrial function, and respirometry, alongside traditional clinical endpoints, such as energy expenditure, heat production, anthropometric measurements, and glycemic control. Similarly, it is essential to include diverse populations, encompassing both sexes, various age groups (children, adults, and older adults), and metabolic conditions (obesity, type 2 diabetes, and diabesity), as well as considering other physiological states such as pregnant and/or breastfeeding women. This will improve the understanding of the efficacy and safety profile of these therapies in different patient subgroups and bring them closer to clinical implementation. Furthermore, future research must take into consideration (i) the optimal dosage and duration, (ii) the administration method, and (iii) the correct treatment time period.

Meanwhile, no plausible pharmacotherapy is currently available in clinical trials to directly target obesity through NST. Currently, although some potential candidate drugs are available, data on their thermogenic effect on humans is scarce; hence, translating basic data to clinical trials targeting different human populations is still needed.

Given that obesity is intimately linked to excessive ROS production, which plays a pivotal role in its pathogenesis, it could be of great interest to combine antioxidant therapies with weight loss strategies. Melatonin stands out among antioxidants due to its dual role, providing antioxidant and anti-inflammatory protection while also acting as a metabolic regulator. Further pharmacological strategies are needed to provide the expected metabolic benefits of increased energy expenditure, reduced body fat, and improved metabolic health, but also reduced oxidative stress damage, without clinically significant side effects. Seemingly, the dual thermogenic capacity of melatonin on muscle and adipose-mediated NST and its beneficial effects on body weight and metabolic health, along with its antioxidant properties and safety profile, make melatonin a plausible therapeutic target and the most optimal clinical candidate for treating obesity and its devastating complications in humans. Well-designed controlled clinical trials with long follow-up periods that take into consideration the dose and time of day of administration are required to assess the potential thermogenic effect of melatonin in obese/overweight individuals and its impact on body weight and metabolic profile, as well as its pharmacological safety in different human populations with various physiological conditions.

Practical challenges, such as standardizing drug doses, administration methods, and the correct treatment period, as well as standardizing nutritional interventions, need to be carefully addressed in order to optimize clinical applicability. In addition, melatonin stands out as a particularly promising candidate due to its multifaceted role in mitochondrial protection, metabolic regulation, antioxidant capacity, and sleep improvement, all of which are critical for patients with diabesity, who often suffer from sleep disorders.

Although the individual intervention trials of thermogenic agents reviewed in this paper are necessary to elucidate their efficacy, it would be interesting for future clinical trials to prioritize a multi-target approach, combining physiological interventions, such as physical exercise and fasting, with nutritional interventions based on hypocaloric diets enriched in thermogenic food-derived bioactive compounds, and pharmacological agents such as melatonin, which has both thermogenic and antioxidant properties. As each stimulus independently activates thermogenesis by different mechanisms, their combined effect could be greater than the sum of individual responses. This integrative approach could synergistically enhance NST activation and metabolic improvements beyond isolated interventions, representing an effective therapeutic approach in the struggle against obesity and related diseases.

## Figures and Tables

**Table 1:** Differential characteristics of the white, brown, and beige adipose tissue types.

Characteristics		White Adipose Tissue 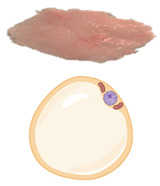	Brown Adipose Tissue 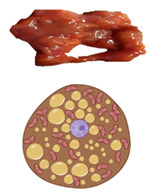	Beige Adipose Tissue 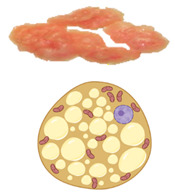
Lipid morphology		Single large droplet	Small multilocular droplets	Small/Medium multilocular droplets
Mitochondrial content		Low	Very high	High
UCP1 protein expression		−	+++	++
Vascularity		Low	Abundant	High
Location	Rodents & human infants	Intra-abdominal (Visceral) Subcutaneous	Interscapular Axillary Perirenal	Inguinal (rodents)
	Adult humans	Intra-abdominal (Visceral) Subcutaneous	Cervical (Deep neck)	Supraclavicular Pericardiac Paravertebral Perirenal
Function		Energy storage as triglycerides	Heat production (Non-shivering thermogenesis)	Thermogenic potential

Adipose tissues’ images were taken from our experimental diabesity model, the Zücker Diabetic Fatty rat.

## Data Availability

Not applicable.

## Abbreviations

The following abbreviations are used in this manuscript:

ADP	Adenosine diphosphate
Akt	Protein kinase B
AMPK	5′ adenosine monophosphate-activated protein kinase
ATP	Adenosine triphosphate
β3-AR	β3 adrenergic receptor
BAT	Brown adipose tissue
bAT	Beige adipose tissue
BMP7	Bone morphogenetic protein 7
Ca^2+^	Calcium
CaMKII	Ca^2+^/calmodulin-dependent protein kinase II
CD36	Cluster of differentiation 36
DHA	Docosahexaenoic acid
DKO	Double knockout
EPA	Eicosapentaenoic acid
ERα	Estrogen receptor α
FGF21	Fibroblast growth factor 21
FNDC5	Fibronectin type III domain-containing protein 5
FXR	Farnesoid X receptor
GH	Growth hormone
GHR	Growth hormone receptor
GIPR	Glucose-dependent insulinotropic polypeptide receptor
GLP-1	Glucagon-like peptide 1
GLUT4	Glucose transporter type 4
GP	Grain of Paradise
GPx	Glutathione peroxidase
GR	Glucagon receptor
HFD	High-fat diet
IF	Intermittent fasting
KO	Knockout
mPTP	Mitochondrial permeability transition pore
MT	Melatonin receptor
NRF1/2	Nuclear respiratory factor 1/2
NST	Non-shivering thermogenesis
OXPHOS	Oxidative phosphorylation
PGC1α	Peroxisome proliferator-activated receptor gamma coactivator 1-alpha
PKA	Protein kinase A
PPAR	Peroxisome proliferator-activated receptor
PRDM16	PR/SET domain 16
PUFA	Polyunsaturated fatty acid
RyR	Ryanodine receptor
SCFA	Short-chain fatty acids
SERCA	Sarcoplasmic/endoplasmic reticulum Ca^2+^-ATPase
SGLT2	Sodium-glucose linked transporter 2
SIRT	Sirtuin
SKM	Skeletal muscle
SLN	Sarcolipin
SOD2	Superoxide dismutase 2
sWAT	Subcutaneous white adipose tissue
T2DM	Type 2 diabetes mellitus
T3	Triiodothyronine
TH	Thyroid hormone
THR	Thyroid hormone receptor
TRP	Transient receptor potential
TRPA1	Transient receptor potential A1
TRPM8	Transient receptor potential cation channel melastatin 8
TRPV1	Transient receptor potential vanilloid 1
UCP	Uncoupling protein
vWAT	Visceral white adipose tissue
WAT	White adipose tissue
